# Marie Krogh's contributions to the study of thyroid physiology and pathophysiology

**DOI:** 10.1113/EP092572

**Published:** 2025-02-25

**Authors:** Per Karkov Cramon, Mathias Loft, Ronan M. G. Berg

**Affiliations:** ^1^ Department of Clinical Physiology and Nuclear Medicine Copenhagen University Hospital – Rigshospitalet Copenhagen Denmark; ^2^ Department of Medical Endocrinology Copenhagen University Hospital – Rigshospitalet Copenhagen Denmark; ^3^ Department of Clinical Physiology and Nuclear Medicine Copenhagen University Hospital – Bispebjerg Hospital Copenhagen Denmark; ^4^ Department of Clinical Medicine, Faculty of Health and Medical Sciences University of Copenhagen Copenhagen Denmark; ^5^ Neurovascular Research Laboratory, Faculty of Life Sciences and Education University of South Wales Pontypridd UK

As recently recounted in *Experimental Physiology*, Marie Krogh (1874–1943; born Birte Marie Jørgensen; Figure 1) had a tremendous impact through her research in diverse areas, including respiratory physiology, endocrinology, pharmacology and nutrition (Berg, 2024). Here, we would like to highlight her extensive studies on the physiology and pathophysiology of the thyroid gland that engaged her clinical and scientific efforts through most of her career.

**FIGURE 1:**
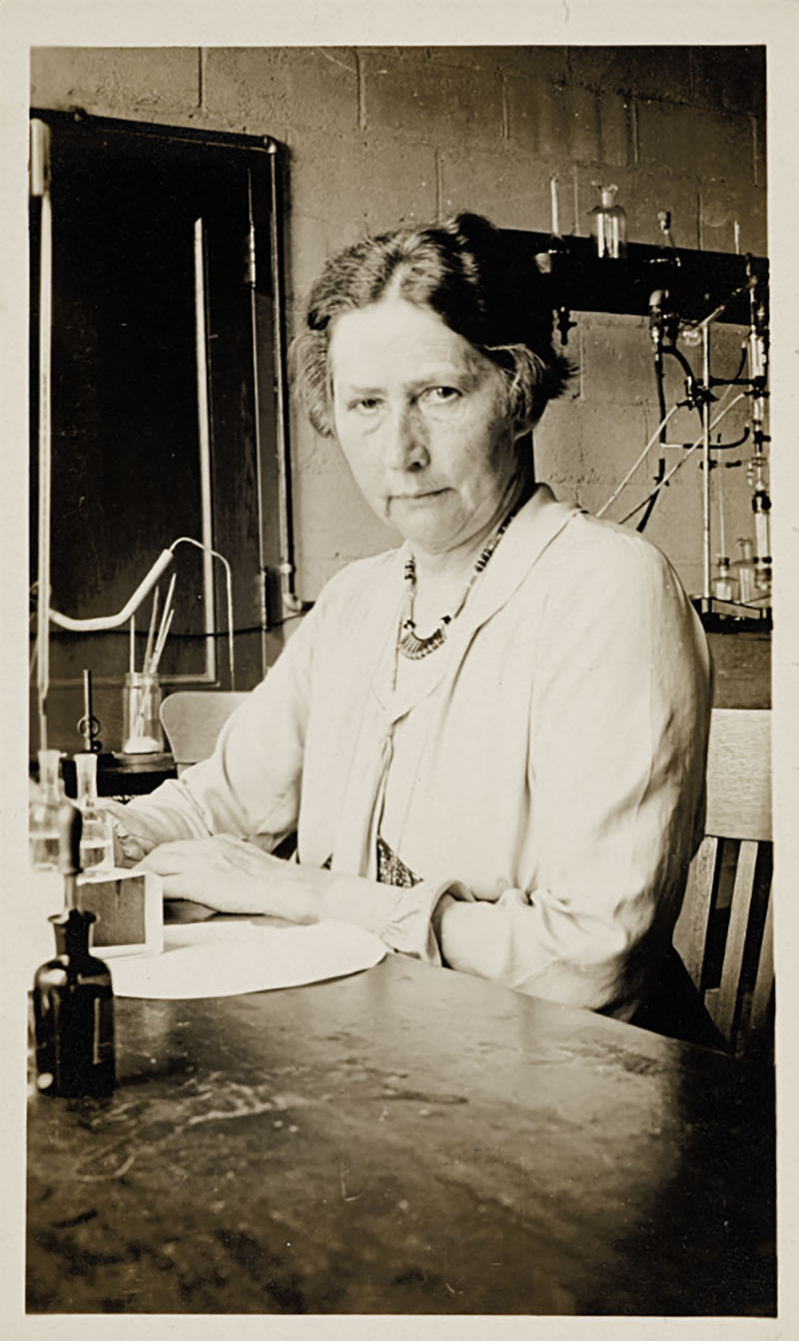
Marie Krogh (1874–1943) in her laboratory in the Rockefeller Institute, Copenhagen ∼1940. Photograph available from The Royal Danish Library, kindly provided by Hanne Sindbæk. Copyright: public domain.

Before Marie Krogh's work on the single‐breath technique for measuring the pulmonary diffusing capacity for carbon monoxide was published in *The Journal of Physiology* (Krogh, 1915), it formed the basis of her doctoral thesis, which earned her the higher (Doctor of Medical Science) degree after a public defence on 28 May 1914. Present in the audience was Hans Christian Joachim Gram (1853–1938), notably famous for inventing Gram staining, a standard technique to classify bacteria and make them more visible under a microscope. Gram was Professor of Medicine at Department A, a Department of General Medicine, at the recently founded Rigshospitalet in Copenhagen. As part of her doctoral studies, Marie Krogh had studied patients with lung disease from Gram's department. Soon after her defence, she secured full‐time clinical tenure there, while simultaneously running her own private clinical practice. Over the years, Gram had developed a keen interest in thyroid diseases. Without digressing too far, it is worth noting that Gram is credited with one of the earliest examples of clinically diagnosing an endocrine disorder from ancient art (Riva et al., 2020). He was particularly keen on diagnosing various thyroid diseases, including goitre and Basedow's disease with exophthalmos, for use in his Danish textbook on thyroid disease (Gram, 1911). Gram identified these conditions in different Roman busts at the Ny Carlsberg Glyptotek in Copenhagen (Figure 2).

**FIGURE 2:**
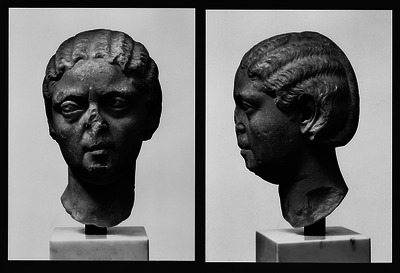
Thyroid disease in ancient art. The marble bust portrays a Roman woman with puffy eyelids and bulging eyes (exophthalmos), in addition to slight goitre, which Marie Krogh's clinical mentor, Professor Hans Christian Joachim Gram (1853–1938), interpreted as evidence of Basedow's disease. Printed with permission from the Ny Carlsberg Glyptotek in Copenhagen.

Patients with thyroid disease soon sparked Marie Krogh's interest. This was during a time when, largely owing to the efforts of Edward Sharpey‐Schafer (1850–1935; born Edward Albert Schäfer), the founding editor of *Experimental Physiology* (initially titled *Quarterly Journal of Experimental Physiology*) (Bailey et al., 2023), endocrinology was emerging as an independent field. Sharpey‐Schafer was a polymath who made numerous contributions to physiology, including the discovery of adrenaline with George Oliver (1841–1915) (Oliver & Schäfer, 1895), landmark studies on the adrenal and pituitary glands, and coining the term ‘endocrine’ for the secretions of ductless glands. He also introduced the term ‘insulin’, derived from the Latin *insula* (‘island’), when he described in detail the hormone produced by pancreatic islets, capable of controlling glucose metabolism (Schäfer, 1916). In fact, it was his book *The Endocrine Organs: An Introduction to the Study of Internal Secretion* that defined endocrinology as an independent field within physiology (Schäfer, 1916), and furthermore, the *Quarterly Journal of Experimental Physiology* contributed significantly to this new field gaining prominence in physiology, particularly after World War I (Borell, 1978).

At Rigshospitalet, Marie Krogh observed that patients with hyperthyroidism lost weight, whereas those with hypothyroidism gained weight, despite seemingly normal diets in both conditions. These findings aligned with several well‐known studies, which suggested prominent effects of thyroid hormones on metabolism (Magnus‐Levy, 1895), although methods for measuring metabolism in previous studies had several inherent flaws. Along with her husband, August, Marie Krogh had previously studied metabolism and diet in the Inuit during an expedition to Qeqertarsuaq, Greenland, in 1908. Their research revealed that the Inuit, despite following a high‐protein, high‐fat diet by Western standards, maintained a normal resting metabolism (Krogh & Krogh, 1913). This work led to the development of methods for assessment of resting metabolism with unprecedented accuracy and precision.

While August Krogh and Johannes Lindhard (1870–1947) were simultaneously studying the effects of high‐carbohydrate versus high‐protein and high‐fat diets on exercise capacity and endurance (Berg, 2025), Marie Krogh began investigating whether patients with thyroid diseases exhibited normal resting metabolism. Her primary objective was to develop a method to improve the diagnosis of patients with mild to moderate Basedow's disease by demonstrating elevated resting metabolism. Over the next 2 years, she obtained measurements from a cohort of patients with the techniques she and August Krogh had developed in Greenland. She was, however, greatly surprised by the results from two cases, which were so unequivocal that she decided to publish them almost instantly (Krogh, 1916). One patient with severe Basedow's disease exhibited a resting metabolism 80% above normal, whereas another patient with severe hypothyroidism showed only 40% of normal resting metabolism! Because normal EMG results ruled out subclinical skeletal muscle contraction, Marie Krogh concluded that the observed changes in metabolism were not caused by overt muscle activity. Instead, she proposed that thyroid hormone affected resting metabolism through effects on resting muscle tone, either because thyroid hormone triggered neural efferent output to the muscle or from a more direct chemical action of the hormone on muscle tissue (Krogh, 1916). To clarify this further, she conducted experiments on anaesthetized frogs at her own dedicated workspace within her husband's increasingly internationally renowned Zoophysiological Laboratory. In these experiments, she infused a preparation made from dried thyroid tissue into the stomach and observed an increase in oxygen metabolism. However, when the nerves to the extremities were severed concurrently, the increase in oxygen uptake was reduced. From these findings, she concluded that the metabolic effects of thyroid hormone were not caused by a direct chemical action on the muscle cells but must instead be mediated neurally (Krogh, 1916).

Today, it is well established that thyroid hormones affect basal metabolic rate through multiple steps and a wide range of metabolic processes across all the tissues in the body (Mullur et al., 2014). These effects occur through both genomic and non‐genomic mechanisms. The former involves thyroid hormone binding to nuclear thyroid receptors, which interact with thyroid response elements to regulate the expression of various metabolically relevant target genes. Non‐genomic effects include modifications of intracellular signalling pathways, for example, via phosphorylation and activation of kinase pathways involved in metabolic regulation. Furthermore, thyroid hormones indirectly influence basal metabolic rate through interactions with the sympathetic nervous system and by affecting adipokine and neuropeptide regulation of the hypothalamic–pituitary–thyroid axis, in addition to adaptive thermogenesis. Thus, Marie Krogh's provisional conclusion that the effect of thyroid hormone on resting metabolism is mediated through a neural pathway is, in retrospect, somewhat simplistic. She was now on a trajectory towards thyroid physiology and endocrinology that would shape the rest of her career. She continued to measure resting metabolism in her patients at Rigshospitalet. As she later described: ‘I didn't have a fixed setup but carried a gas meter, mixing chamber, and stand with containers around the wards, and then took the containers with exhaled air samples home for analysis. It was quite a hassle, and determining the metabolism of just one patient daily took a long time’ (Sindbæk, 2022). She thus began bringing patients to the Zoophysiological Laboratory and, later, also to her own clinical practice. Ultimately, she reported findings from a series of bedridden patients with thyroid disease, comprising 23 individuals with manifest or suspected hyperthyroidism (Krogh, 1920). Her results demonstrated that the severity of hyperthyroidism was associated with an increase in resting metabolism. Furthermore, she showed that bed rest combined with intense nutritional supplementation to increase weight, a common treatment for hyperthyroidism at the time, did not reduce resting metabolism as expected but, rather, increased it. In contrast, radiation therapy of the thyroid caused the anticipated reduction in resting metabolism (Krogh, 1920).

Despite its imminent clinical implications, Marie Krogh's time‐consuming technique for measuring resting metabolism remained unsuitable for clinical purposes, with each measurement taking several hours to perform. To help her, August Krogh developed a new device, the tilting spirometer, which made the analysis of exhaled air much less cumbersome by integrating the measurement of respiratory volumes and their gaseous composition into a single apparatus (Krogh, 1922). Now, Marie Krogh could obtain results as soon as the patient had been examined and use them for clinical diagnosis. During these same years, Marie Krogh was diagnosed with diabetes mellitus, which, at the time, was untreatable. She was followed discreetly by the physician Hans Christian Hagedorn (1888–1971) in his private clinical practice, and a close friendship developed between the Kroghs and Hagedorn. Soon, Marie Krogh managed to inspire Hagedorn with her enthusiasm for thyroid physiology. Their correspondence from these years indicates that they began collaborating on the development of a drug designed to mimic the effects of thyroid hormone, with the aim of increasing resting metabolism in patients with hypothyroidism (Sindbæk, 2022).

In the late 1920s, Marie Krogh began collaborating with a young and ambitious Danish pathologist, Harald Okkels (1878–1970), later to become professor of anatomy at the University of Copenhagen. Okkels had a particular interest in thyroid histopathology and was working on an impressively large collection of thyroid specimens from patients with various types of goitres (Okkels, 1932). One of their first joint ventures was to identify the best animal model for experimental toxic goitre, a condition induced by repeated intraperitoneal injections of anterior pituitary extract, which closely resembled Basedow's disease. The induced changes in thyroid morphology included cell swelling, foamy cytoplasm, increased mitosis, and a growth in the mass of the Golgi apparatus, with thyroid tissue proliferation resembling parenchymatous goitre in many respects. Such changes were observed in rabbits, guinea‐pigs and other species, including rats, mice and ducks. However, there was uncertainty about which animal model was the most suitable for experimental studies on thyroid disease. In experiments to be published several years later (Krogh & Okkels, 1936), Krogh and Okkels compared rabbits and guinea‐pigs and concluded that rabbits were unsuitable as an experimental toxic goitre model, because their thyroid glands exhibited lesser histopathological responses and far more variable changes in response to anterior pituitary extract in comparison to guinea‐pigs. Furthermore, they noted that the thyroid glands of untreated rabbits, in normal conditions, displayed considerable structural variability influenced notably by season, temperature and diet (Krogh & Okkels, 1936).

Marie Krogh and Okkels proceded with studies on thyroid physiology using their now established guinea‐pig model. Using males, they demonstrated that morphological changes in the thyroid gland, induced by subcutaneous injections of anterior pituitary gland extract, were accompanied by increased metabolism (Krogh & Lindberg, 1932). They concluded that the responsible compound (later identified as thyroid‐stimulating hormone) was distinct from anterior pituitary sex hormones, because the administration of these hormones, isolated from urine, did not produce any structural changes to the thyroid gland (Krogh & Lindberg, 1932). Furthermore, they found that oral administration of the anterior pituitary extract failed to induce changes in either metabolism or thyroid gland structure, showing the instability of the compound in the stomach (Krogh & Lindberg, 1932). In a subsequent study, they compared the effects of oral administration of thyroxine (T_4_) and dried thyroid tissue on resting metabolism in guinea‐pigs (Krogh et al., 1932). Much to their surprise, they found that, when adjusted for iodine content, the activity of thyroxine was approximately two‐thirds that of dried thyroid tissue. They interpreted this provisionally to reflect that dried thyroid tissue was absorbed more readily from the gastrointestinal tract than thyroxine.

Marie Krogh and Okkels went on to investigate the metabolic effect of iodine in guinea‐pigs with experimental hyperthyroidism. In animals continuously stimulated with anterior pituitary extract, the resting metabolism returned to near normal levels after 1 week of daily iodine administration (Okkels & Krogh, 1933) In this manner, they demonstrated the metabolic effects of the later‐discovered Wolff–Chaikoff effect: ingestion of large amounts of iodine inhibit organification of iodide in the thyroid gland and thus inhibit thyroid hormone synthesis (Wolff & Chaikoff, 1948). In contrast, iodine treatment did not normalize metabolism in guinea‐pigs fed with dried thyroid tissue. They applied powerful localized X‐ray irradiation to the pituitary glands of guinea‐pigs and observed lower resting metabolism after some weeks. Correspondingly, the excised thyroids had pronounced morphological changes indicative of reduced activity (Okkels & Krogh, 1933). This experiment elegantly demonstrated how the thyroid gland is dependent on thyroid‐stimulating hormone to maintain normal function and structure.

Marie Krogh and Okkels now turned their attention to chemical properties of the thyroid‐stimulating hormone, including different purification methods. They found that thyroid‐stimulating hormone could be extracted and precipitated from anterior pituitary extract with alcohol (Okkels & Krogh, 1933) Furthermore, they succeeded in obtaining a protein‐free preparation with higher yield. They tried to purify thyroid‐stimulating hormone from urine samples from patients with exophthalmic goitre. Only one of the guinea‐pigs given urine extract demonstrated both a metabolic and a histocytological effect (Okkels & Krogh, 1933). This is in line with what we now know, which is that thyroid‐stimulating hormone is completely suppressed in patients with untreated exophthalmic goitre.

Marie Krogh died from breast cancer in 1943. At her deathbed, August promised to write up her final results on a series of experiments in the guinea‐pig model. Although many probably consider her 1915 paper on the single‐breath technique for measuring pulmonary diffusing capacity her magnum opus (Krogh, 1915), Marie Krogh herself would probably have pointed to this posthumously published work on thyroid physiology, which represented her final aspirations to develop a thyroxine‐like drug (Krogh & Lindberg, 1945). She started this series of experiments when a new orally administered drug for hypothyroidism, Elityran, produced from partly purified thyroid extract, was developed with claims of being up to 10 times more potent than subcutaneously injected thyroxine. Elityran was considered by some to be a potential ‘wonder drug’ for inducing weight loss, even in individuals with normal thyroid function, because it was thought to evade side‐effects, such as increased heart rate, increased respiratory rate, thirst, hair loss and restlessness. This probably piqued Marie Krogh's interest, because her and Hagedorn's previous efforts to develop a drug for increasing resting metabolism in individuals with hypothyroidism had proved unsuccessful. By using the guinea‐pig model, Marie Krogh demonstrated that when orally administered thyroxine or preparations made directly from thyroid tissue, including Elityran, were dosed to cause a similar increase in resting metabolic rate, the occurrence of side‐effects closely followed the elevation in metabolic rate (Krogh & Lindberg, 1945). In accordance with her previous findings (Krogh & Lindberg, 1935), a notably higher dose of thyroxine than the thyroid tissue‐based agents was still required to achieve the same increase in metabolic rate. However, Marie Krogh also showed that when ingested orally, thyroxine had the same metabolic effect as when administered by subcutaneous injection (Krogh & Lindberg, 1945). It was thus clear that the lesser metabolic effect of thyroxine in comparison to thyroid tissue‐based agents was attributable to better absorption of the latter in the gastrointestinal tract, but the underlying mechanism remained obscure for the time being. Unbeknownst to Marie Krogh, her findings demonstrated the physiological effects of triiodothyronine (T_3_), which is also present in thyroid tissue and possesses three to four times greater potency than thyroxine. However, triiodothyronine was not discovered formally until several years later (Gross & Pitt‐Rivers, 1952).

Although it is uncertain whether Marie Krogh shared Gram's passion for Roman art, her dedication to the study of the thyroid gland was undeniable, but despite her and Hagedorn's efforts, she never succeeded in developing an effective thyroxine‐like drug to increase resting metabolism that was suitable for clinical use. Indeed, despite substantial international efforts during her lifetime to develop weight‐loss medicines that acted by increasing resting metabolism, these attempts were never successful. Nevertheless, these efforts coincided with a historical surge in the study of endocrinology that also saw the discovery of insulin. In fact (again, without digressing too far), it was August Krogh who acquired the rights to produce insulin from Frederick Banting (1891–1942) and Charles Best (1899–1978) in Toronto, such that Marie Krogh became one of the first diabetes patients to receive insulin. This resulted in the founding of Nordisk Insulin Laboratorium, which later merged with Novo Industri in 1989 to form the present‐day pharmaceutical empire Novo Nordisk A/S. Ultimately, Novo Nordisk A/S succeeded in developing the long‐sought ‘wonder drug’ for inducing weight loss and treating diabetes, not through thyroid‐like mechanisms, but rather by mimicking the effects of the gut hormone glucagon‐like peptide 1 on appetite and blood glucose homeostasis. This development clearly ensured Novo Nordisk A/S an international impact and sphere of influence, both scientifically and on the stock market, at times reminiscent of that of ancient Rome! Arguably, however, it represents a provisional peak in the clinical–translational research tradition within endocrinology, conceived by the Kroghs, Hagedorn, Gram and Sharpey‐Schafer.

## AUTHOR CONTRIBUTIONS

Per Karkov Cramon: first draft, revisions. Mathias Loft: first draft, revisions. Ronan M. G. Berg: conception, first draft and revisions. All authors approved the final version of the manuscript and agree to be accountable for all aspects of the work in ensuring that questions related to the accuracy or integrity of any part of the work are appropriately investigated and resolved. All persons designated as authors qualify for authorship, and all those who qualify for authorship are listed.

## CONFLICT OF INTEREST

No authors have any conflict of interest to declare.

## FUNDING INFORMATION

None.
